# Epidemiology of Type 3 Poliovirus AFP Cases in Israel between 1973 and 1988: Whole Genome Sequencing of RNA Extracted Directly from Archived Stocks to Avoid Re-Culturing Neurovirulent Wild Poliovirus

**DOI:** 10.3390/vaccines10122154

**Published:** 2022-12-15

**Authors:** Lester M. Shulman, Majid Laassri, Rachel Handsher, Tatiana Zagorodnyaya, Danit Sofer, Merav Weil, Ella Mendelson, Konstantin Chumakov

**Affiliations:** 1 Central Virology Laboratory, Public Health Service Laboratories Israel Ministry of Health at Sheba Medical Center, Tel Hashomer, Ramat Gan 52621, Israel; 2 Department of Epidemiology and Preventive Medicine, School of Public Health, Sackler Faculty of Medicine, Tel Aviv University, Tel Aviv 69978, Israel; 3 US Food and Drug Administration, Center for Biologics Evaluation and Research, Silver Spring, MD 20993, USA

**Keywords:** type 3 poliovirus, whole genome sequencing, AFP (acute flaccid paralysis), recombination

## Abstract

Background: Poliovirus post-eradication containment of wild-type 2 poliovirus (PV2) requires the destruction of all materials containing, or potentially containing, PV2. Acute flaccid paralysis (AFP) cases in Israel between 1973 and 1988 were caused by all three serotypes; thus, isolates from cases and case-contacts were either PV2 or potentially contaminated with PV2. Aims: To provide a proof-of-concept that whole genome sequences (WGS) of wild-type 3 poliovirus (PV3s) could be salvaged from the RNA extracted directly from archived poliovirus stocks avoiding re-amplification of neurovirulent viruses, we link WGSs to case histories and determine the phylogenetic relationships among the PV3s. Methods: Data retrieved from 427 poliovirus-positive cases reported between 1973 and 1988 identified 85 PV3-associated cases. A total of 71 archived PV3 isolates were available from PV3-positive cases and contacts. WGSs were obtained by NGS from cDNA libraries constructed from RNA extracted directly from archived viral stocks. Sequences were subjected to phylogenetic analysis and linked to case data. Results: WGSs were successfully constructed for 55 isolates. Phylogenetic analysis revealed the circulation of seven lineages of PV3. One lineage, with 23 isolates, presented as an outbreak of six-year duration. Isolates from six other lineages were consistent with subsequent separate introductions, sporadic cases, and limited transmission. Recombinant vaccine-like PV3 recombinants were isolated from some cases. Conclusions: Whole or near-whole genome sequence information, obtained from RNA extracted directly from the archived material, safely provided detailed genetic information linked to patient data from a time when limited sequence information was previously available and revealed the pattern of transmission of wild PV3 in Israel.

## 1. Introduction

There are three serotypes of poliovirus, poliovirus type 1 (PV1), type 2 (PV2), and type 3 (PV3). Two types of poliovirus vaccine were developed and used globally, since the 1950s to prevent infections with the three serotypes of poliovirus, as well as the subsequent development of poliomyelitis (acute flaccid paralysis, AFP, resulting from damage to Motor neurons and denervation of muscles in a small minority of individuals infected with poliovirus). One vaccine contained inactivated polio virus strains (IPV) [1], and the other contained live attenuated poliovirus strains (OPV) [2]. Both were trivalent, e.g., they contained a representative strain of each of the three serotypes.

Between 1957 and 1987, various preparations and schedules of OPV were used in Israel (exception: two of fourteen health districts exclusively used IPV between 1982 and 1987) [3,4]. A supplementary immunization of almost all individuals up to the age of 40 in 1988 with a dose of OPV in response to an outbreak of wild-type 1 poliovirus that had begun in 1987 prevented further infections and AFP caused by any of the three wild poliovirus serotypes [3,4,5]. 

Wild-type 2 poliovirus (WPV2) was declared eradicated in 2015 [6,7]. This, in turn, triggered two events. The first was a globally coordinated replacement of the trivalent oral polio vaccine (OPV), which contained representative attenuated strains of Type 1, Type 2, and Type 3 poliovirus, with bivalent polio vaccine (bOPV, oral polio vaccine that only contains representative attenuated strains of Type 1 and Type 3 poliovirus) [8]. The second event was the initiation of phase 2 of the global action plan (GAP III) [9]. Annexes 2, 3, and 6 of GAP III require the containment of all type 2 polio viruses in polio essential facilities (PEFs) and the destruction of all Type 2 polioviruses present in non-essential polio facilities or their transfer from the non-PEFs to PEFs [9]. Most polio laboratories in the WHO global polio laboratory network (GPLN), including The Israel National Center for Polio and non-Polio Enteroviruses, chose to remain non-PEFs, rather than undergo the difficult and expensive procedure of becoming annually certified as a PEF by the WHO. 

The Israeli laboratory’s archived collection of 257 wild polioviruses that were isolated from clinical AFP cases and contacts between 1973 and 1988 that were transferred to a PEF at the FDA in White Oaks, MD, for whole genome sequencing (WGS). Whole genome sequences can be generated using next generation quenching (NGS) techniques. NGS employs a number of different strategies to sequence the equivalent of a whole genome in a single reaction without the need to design specific primers for the poliovirus isolate of interest [10]. The NGS strategy used in this study is based on fragmentation of RNA into short 150–250 nt segments, converting the fragments into cDNA using random primers, ligating short sequences of DNA containing targets for a single set of primers to the ends of all cDNA fragments, amplifying each fragment into separate clusters on a flow cell, and then separately determining, in parallel, for all clusters, which of four fluorescently-labeled nucleotides are added to the growing complimentary strand during successive cycles of elongation. Contigs, the formation of a longer sequence from overlapping shorter sequences, representing whole genome sequences are generated in silico by either aligning short sequences reads to reference sequences or by the de novo assembly of overlapping sequences. However, VP1 sequences remain the standard for molecular intratypic differentiation (ITD) of polioviruses.

For safety, polioviruses RNA for sequencing was extracted directly from these samples without any need to grow high titers of neurovirulent viruses in tissue cultures. Genetic and phylogenetic analysis, based on the WGS of representative isolates of PV3 presented here, together with a summary of the demographic and clinical data associated with AFP cases and/or contacts with PV3-positive stools, shed light on the epidemiology of poliovirus infections in Israel between 1973 and 1988. WGS also significantly increased the existing database of sequences of the other three capsid proteins, non-structural genes, and non-coding and regulatory region PV3s that circulated in the Middle East during these years.

## 2. Materials and Methods

### 2.1. AFP Cases and Contacts

The Central Virology Laboratory of the Israel Ministry of Health investigated 1020_T2_._01_ AFP cases between 1973 and 1988. The annual incidence of AFP cases and estimated AFP rate per 100,000 children ≤15 years old is presented in Table 1. The list of isolates and AFP cases described in this manuscript is presented in Table 2. Note: Of the 1020_T2_._01_ AFP cases, 427_T2_._02_ cases reported poliovirus-positive stools. Seventy-three_T2_._04_ AFP cases had PV3(+) stools. There were 93_T2_._08_ individuals with PV3(+) stools. This includes stools from PV3(+) cases and from PV3(+) contacts of PV3(+) cases and PV3(−) cases with ≥1 PV3(+) contact. The Global Polio Surveillance Action Plan, 2018–2020, of the Polio Global Eradication Initiative defines contacts of AFP cases as follows:

*“AFP contact sampling is the collection and testing of stool samples from contacts of AFP cases. A contact of an AFP case is defined as a child (preferably younger than five years of age) who likely had direct contact with the AFP case in the week prior to the onset of paralysis and/or in the two-week period after onset of paralysis. …AFP contact sampling is done to increase the sensitivity of the surveillance system to detect circulating polioviruses (wild and/or vaccine-derived) and, during an outbreak, to gain a better understanding of the geographic extent of the transmission.”*

https://polioeradication.org/tools-and-library/resources-for-polio-eradicators/gpei-tools-protocols-and-guidelines/, last accessed 1 September 2022.

Demographic data (Table 3) associated with AFP cases and/or contacts with PV3-positive stools was obtained retrospectively from summarized case reports maintained by the Central Virology Laboratory. 

### 2.2. RNA Extraction from Archived PV3-Positive Isolates for Whole Genomic Sequencing (WGS)

RNA was extracted directly from 1 mL aliquots of archived PV3-positive isolates for two reasons: (1) for safety reasons to avoid the necessity for growing high titter stocks of neurovirulent wild polioviruses; and (2) the unknown viability of 71 archived stocks of PV3s isolated from stools of cases or contact that had been stored at −20 °C for many years. RNA extraction was performed on 1 mL aliquots of archived cell culture supernatant from the earliest passage that had been archived using a MagNA Pure LC2.0 Automatic extractor with MagNA Pure LC Total Nucleic Acid Isolation Kit-High Performance (Roche Diagnostics, Indianapolis, IN, USA) and eluted into 50 µL of elution buffer, according to manufacturer’s instructions. PV3-positive RNA suitable for whole genome sequencing was extracted from 55_T2_._10_ samples. The remaining 16 samples were not subjected to RNA extraction, did not yield RNA suitable for WGS, or only yielded partial sequences of insufficient length to confirm the poliovirus serotype or use for phylogenetic analysis.

### 2.3. Whole Genome Sequencing (WGS)

cDNA libraries, ranging from 200 to 500 bp in size, were prepared from the 0.5 µg of the total RNA extracted from 53_T2_._11_ archived test tubes of poliovirus isolates from PV3(+) cases plus 2_T2_._12_ isolates from PV3(−)PV(+) cases yielding WGS for 55_T2_._10_ PV3 isolates. Briefly, NEBNext mRNA Sample Prep Master Mix Set 1 (New England BioLabs, Ipswich, MA, USA) was used to prepare RNA libraries, according to the manufacturer’s protocol (NEB), as described by Laasri et al. [11]. RNA was fragmentated by a focused ultrasonicator (Covaris) to generate the fragments of optimal sizes (250–300 nt). cDNA was synthesized using SuperScript III Reverse Transcriptase (Invitrogen, Waltham, MA, USA) and random primers. The cDNA was converted into double stranded cDNA, followed by an end repair procedure (Klenow fragment, T4 polynucleotide kinase, and T4 polymerase) and was ligated to Illumina (San Diego, CA, USA) paired-end (PE) adaptors. A double AMPure bead selection step (Beckman Coulter, Brea, CA, USA) was used for size selection. The DNA libraries were expanded using 15 cycles of PCR, with multiplex indexed primers and purified by magnetic beads (Agencourt AMPure PCR purification system, BeckmanCoulter). Quality and size distribution of the DNA fragments was confirmed on a BioAnalyzer using a high sensitivity kit (Agilent Technologies, Inc., Santa Clara, CA, USA).

Deep sequencing was performed using MiSeq (Illumina), producing paired-end reads 250 nt long, or HiSeq2000 (Illumina) producing 101 nt long paired-end reads. The sequencing reads were analyzed by the in-house ‘swarm’ or High-Performance Integrated Virtual Environment (HIVE) software the DNA fragments [12]. A curated database of 500 reference enteroviruses was used as the template for aligning the short sequences reads to the reference sequences in the database. Contigs containing complete sequences of the coding sequences (P1 which encodes all four capsid proteins and P2 and P3 which encode the nonstructural genes) were generated. Insufficient depth of coverage at either end of the genome made reliable sequence reconstruction of the ends of the 5′ and 3′ coding regions difficult for some of the contigs. In samples where more than one poliovirus was present, the individual consensus sequences were generated using the algorithm described in Karagiannis et al. [13].

Isolates were named as follows: Their typical differentiation (“WPV3” for wild-type 3 poliovirus or “SL3” for Sabin 3-like); the 3-letter country code “ISR” for Israel; the 4-digit year of isolation and two-digit incremental number assigned to indicate the incremental order of the case from which they were isolated during that year; and an uppercase letter indicating the lineage (example: isolate WPV3_ISR_1973_17-A1 is a wild PV3 of lineage A1 and was isolated from the 17th AFP case in 1973). Sub-lineages of isolates from lineage A have an additional number and lowercase letter following the “A”). Genomic sequences were deposited in the GenBank/EMBL/DDBJ database (accession numbers OP137272-OP137324). 

### 2.4. Phylogenetic Analysis

PV3 whole genome sequences were aligned with the Sabin 3 vaccine strain sequence (GenBank access number AY421821) using the Sequencher program v5.4 (Gene Codes Corp., Ann Arbor, MI, USA). Aligned sequences were trimmed in silico to produce aligned sequences of VP1, P1, and the entire coding region (P1-P2-P3) for phylogenetic analysis using the equivalent sequences of Sabin 3 (AY421821) as the outgroups.

Aligned sequences were exported to Clustal x [14], where the sequences were re-aligned, and a neighbor-joining tree created after bootstrapping for 1000 iterations. The resultant trees were visualized using NjPlot v2.3 (http://pbil.univ-lyon1.fr/software/njplot.html last accessed 14 November 2022.) and annotated using Intaglio 3.95 (intag lio@PurgatoryDesign.com; https://pcmacstore.com/en/app/417852764/intaglio%20last%20accessed%2014 last accessed 14 November 2022). Aligned sequences for specific lineages were analyzed for recombination events using the SimPlot program v3.5 (https://sray.med.som.jhmi.edu/SCRoftware/ last accessed 14 November 2022.).

## 3. Results

AFP case reports from 1973–1988 (*n* = 1020_T2_._01_) were examined retrospectively. There were 85_T2_._03_ AFP case reports indicating that PV3 had been detected in stools from the case and/or ≥1 contact. PV3 was isolated exclusively from 73_T2_._04_ cases. The remaining 12 cases had PV3-negative stools, but ≥1 PV3-positive contact (four poliovirus-negative cases with PV3(+) contacts and eight PV1-positive and/or PV2-positive-cases with ≥1 PV3-positive contact). Complete or near-complete whole genome sequences were obtained by NGS for 53_T2_._11_ PV3 isolates had been archived from cases or contacts of the 85_T2_._03_ AFP cases (Table 2 and Table 4). In addition, there was one PV1-positive case report, WPV3_ISR_1974-20_A2a, that yielded a unique WPV3 sequence, instead of a PV1 sequence by NGS. 

**Figure 1:**
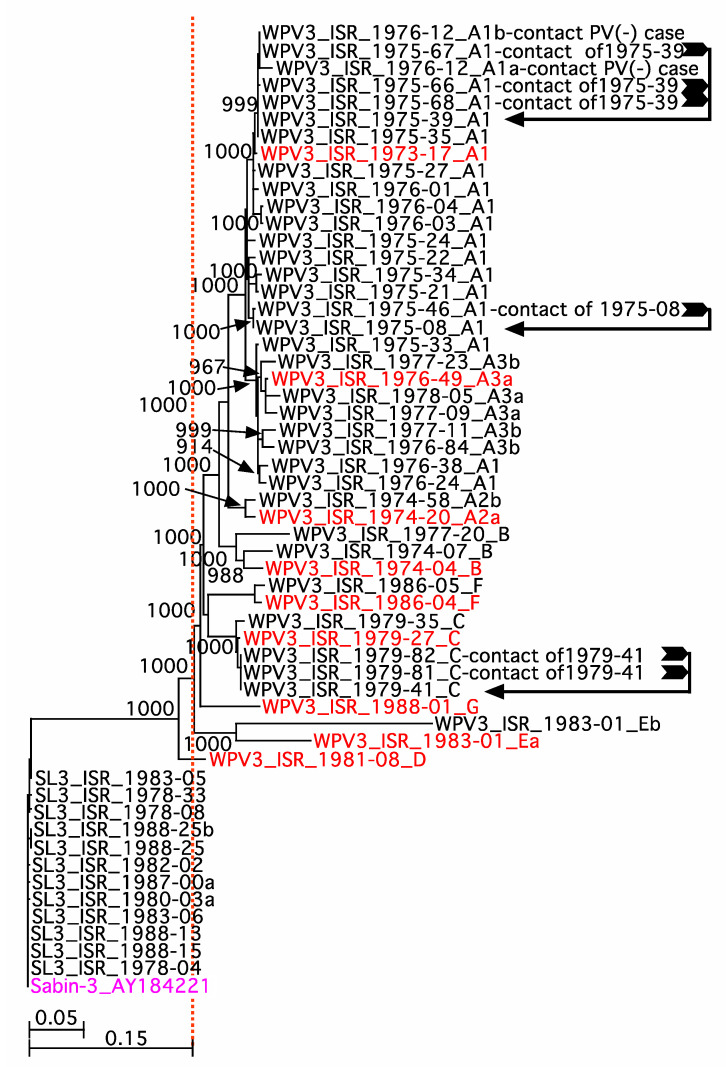
Neighbor-Joining Phylogenetic Tree of the P1 regions of PV3 isolates. The tree was constructed using unweighted pair group method with arithmetic mean (UPGMA). Sabin 3 (text in purple) was set as the outgroup. The vertical dashed line is set at a divergence of 15% from Sabin 1. Bootstrap values (*n* = 1000) are indicated near the internal nodes of the tree. Black arrows indicate the AFP case for an isolate from a contact of that case. The lineages are indicated by the letter or letters, following the year of isolation and the order in which the stool from the AFP case was collected. Text in red indicate the earliest isolate in each lineage.

Of the 55_T2_._10_ sequenced isolates, 43_T2_._13_ isolates from 34_T2_._13_ cases or case-contacts, was classified as wild-type 3 polioviruses, WPV3s, since their VP1 sequence was >15% different from the VP1 sequence of Sabin 3 VP1 (AY421821). These isolates are to the right of the vertical dashed line in Figure 1. The remaining 12_T2_._14_ isolates from 11_T2_._14_ archived samples were classified as Sabin 3-like (SL3), since their VP1 sequence diverged from that of Sabin 3 vaccine strain by less than 1% (sequences to the left of the vertical dashed line in Figure 1. Of the 43_T2_._13_ WPV3 isolates, 35 of43_t2_._13_ isolates were obtained from stools from 34_T2_._13_ AFP cases (two different WPV3 sequences were obtained from case 1983-01), and 8 WPV3 isolates were obtained from stools of contacts (6 from 3 of the sequenced cases and two from poliovirus-negative cases). Of the 12_T2_._14_ SL3s, 1_T2_._16_ (SL3_ISR-1978-33) came from a case reporting PV2 and PV3 isolates, 9_T2_._15_ from PV3 cases, 1_T2_._17_ (SL3-ISR-1988-25) came from a PV3 case that also contained an isolate that yielded an incomplete WPV3 whole genome sequence, and 2 isolates were obtained from one archived sample. 

A bootstrapped, neighbor-joining phylogenetic tree with Sabin 3 (AY411821) as outgroup was prepared for P1 sequences (2634 nt encoding 878 amino acids) (Figure 1). WPV3 isolates sequences from cases and contacts was segregated into 7_T2_._18_ lineages. The lineages of the PV3 isolates were arbitrarily designated with the letters A to G in alphabetical order, corresponding to the year of isolation of the first isolate in the lineage (name in red text in the figure). Simplot analysis of complete open reading frames (6618 nts) encoding the structural and non-structural genes revealed that the sequences in lineage A could be subdivided into three sub-lineages, A1, A2, and A3, and sub-lineages A2 and A3 could be sub-divided into two and three sub-sub-lineages, respectively, based on major sequence differences in their P2-P3 nonstructural gene coding regions, resulting from different recombination events (not shown). A1 case isolates (*n* = 15_T2_._20_) were obtained from stools of non-Jewish Arab patients, 14 from Gaza and 1 from the West Bank/East Jerusalem region. Of the two_T2_._21_ A2 case isolates, both were obtained from non-Jewish Arab patients, one was from Gaza, and the other from the West Bank. For the 6_T2_._22_, the A3 case isolates were also obtained from stools of non-Jewish Arab patients, eight from Gaza, and one from an Israeli health district. 

The presumed recombinant regions within the P2-P3 genomic regions were excised in silico and subjected to an NCBI nucleotide BLAST search [15] of the GenBank nucleotide collection (nr/nt) in an attempt to identify the non-polio enterovirus that supplied the recombinant genome segment. It was not surprising that closest matches were ≤86% homologous with contemporary viruses, due to the paucity of the available sequence data. The exception was that sequences from then P2-P3 region of isolates WPV3_ISR_1986-04_F and WPV3_ISR_1988_01_G both shared up to 90% homology with the WPV3s isolated in Finland in 1984. 

**Figure 2:**
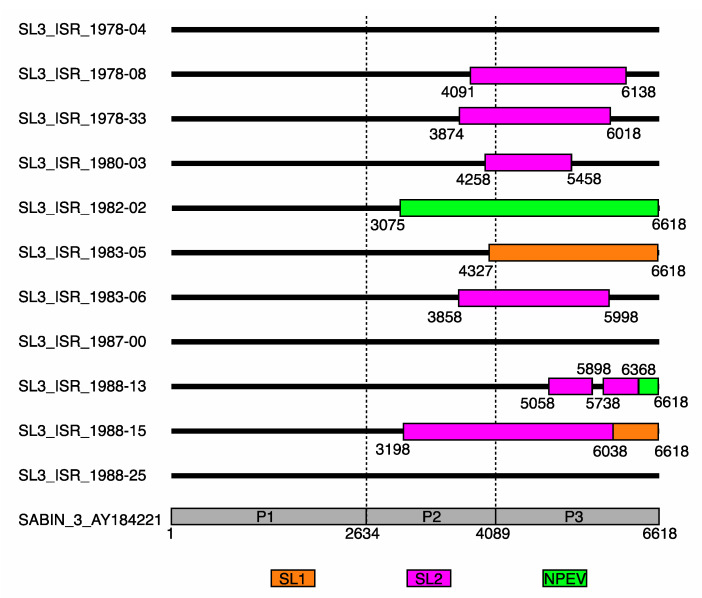
Recombination events in Sabin 3-like polio isolates. The colored rectangles in the P2, P3, and 3′ noncoding regions of the genomes indicate range and the donor of the recombined fragments. The numbers below the rectangles indicate the nucleotide positions at the beginning and ends of the recombined fragments, relative to Sabin 1.

WGS of 8_T2_._23_ of the 12_T2_._14_ isolates from 11_T2_._14_ cases with SL3 VP1 sequences revealed that they had recombined with sequences from OPV1, OPV2, and/or a non-polio enterovirus (NPEV) in their P2 and/or P3 regions (Figure 2). Seven isolates were obtained from Jewish children in Israeli health districts, with two from Arab children whose geographic location was not available and three from cases lacking information.

In silico analysis of nucleotides associated with reversion to neurovirulence, (1) U_472_C in 5′UTR, (2) U_2034_C (VP3 phe_91_ser), and (3) U_2493_C (VP1 thr_6_iso), are presented in Table 5. Four SL3 isolates had fully reverted to neurovirulence, six had reverted at two of three sites, and one had reverted at only one site. Of note, seven were isolates obtained within 30 days of a dose of OPV.

**Figure 3:**
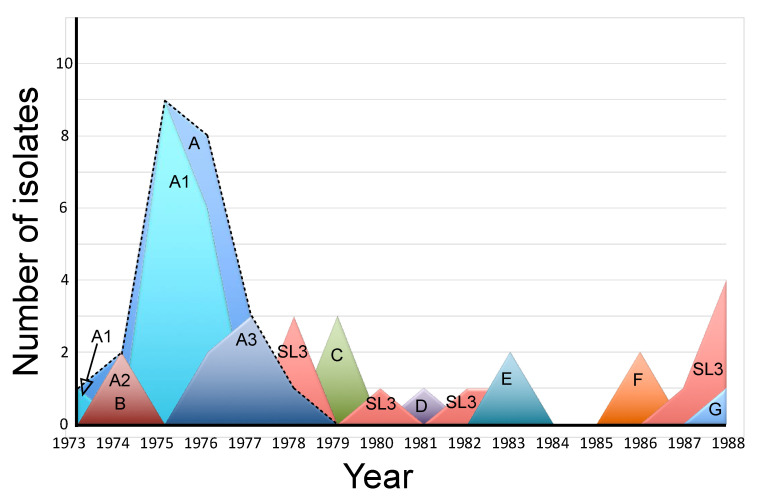
Annual incidence of PV3 lineages. The graph indicates the annual number of sequence-confirmed isolates belonging to the different lineages of type 3 poliovirus isolated from stools of AFP cases in Israel between 1973 and 1988. The letters A-G refer to the lineages in Figure 1. The 23_T2_._19_ isolates of Lineage A fall under the dashed line labeled “A”. A1, A2, and A3 are three sub-lineages within Lineage A.

The annual incidence of the different lineages of WPV3 and SL3 isolates was graphed (Figure 3). Isolates of the A lineage, the lineage with the most isolates, continued to circulate between 1973 and 1978. The remaining lineages appeared sporadically over periods of one to three years from 1974 to 1988. The SL3 isolates appeared throughout the time period investigated. 

The clinical and demographic data presented in Table 6, Table 7, Table 8 were calculated from case report summaries for the following five categories: (1) all 85_T2_._03_ AFP cases where stools from the case and/or ≥ 1 contact contained PV3; (2) all 73_T2_._04_ AFP cases where PV3 was isolated from the case; (3) all 43_T2_._13_ AFP cases with complete WPV3 capsid sequences; (4) the 23_T2_._19_ cases with isolates that mapped to lineage A or a sub lineage of A; and (5) the 11_T2_._14_ cases that had SL3-like isolates. Summarized case reports were reviewed for vaccination histories of PV3-positive individuals. The vaccination histories and the interval between the last OPV dose and appearance of symptoms are presented in Table 6. Clinical symptoms from case reports where they were recorded are summarized in Table 7. Finally, kinetic changes in anti-polio IgG titers after onset of symptoms (seroconversion or ≥ 4-fold increases), when recorded in case reports, are summarized in Table 8.

## 4. Discussion

Our current database of time-dated poliovirus sequences reflects the developments in the field of genome sequencing. The transition from Sanger sequencing, through automation of Sanger sequencing, to next generation sequencing has enabled us to easily generate longer and longer sequences culminating in whole genome sequences. Many of the sequences available for the earliest isolates cover only 250 nucleotides, spanning the junction between capsid protein 1 (VP1) and protease 2a. This segment served as the initial standard for sequence-based intratypic differentiation (ITD), e.g., the characterization of poliovirus isolates as either Sabin strain vaccine-like (SL) or wild (WPV), based on comparison to the same region of the corresponding Sabin vaccine strain [16]. Automation of Sanger sequencing enabled ITD comparisons to shift to the 900–906 nucleotides of the complete VP1 sequence [17]. Isolates were classified as Sabin vaccine strain-like (SL) when VP1 sequences diverged from the Sabin vaccine strain by ≤1% for PV1s and PV3s and ≤0.6% for a PV2, as vaccine-derived poliovirus (VDPV) for divergence between 1–15% or 0.6–15% or wild poliovirus WPV, when differences were in excess of 15% [18]. The concatenation of sequences of overlapping genome segments generated in separate Sanger sequencing reactions using specific primers was a method for generating whole genome sequences. It was labor intensive, and in many cases, required the design of new primers whenever genomic sequences became insufficiently complimentary, due to individual substitutions of individual nucleotides or major differences resulting from genetic re-combinations with other polioviruses and/or non-polio enteroviruses. 

Many laboratories kept collections of un-sequenced or partially sequenced wild polioviruses with linked case reports that were isolated before complete genome sequences could be easily generated by NGS. Containment of type 2 poliovirus (phase 2 of GAP III), invoked when type 2 wild poliovirus [ was declared eradicated in 2015 6,7,9], required the destruction of all samples containing or potentially containing PV2 or their transfer to a polio essential facility (PEF). Many laboratories chose to destroy samples from enteric infections, including those containing type 1 or 3 polioviruses, in addition to those known to contain type 2, since they could not rule out the possibility that they might also have contained some type 2 poliovirus. The Israel National Center for Polioviruses and non-Polio Enteroviruses, instead, chose to transfer aliquots of annotated polioviruses from AFP cases isolated between 1973 and 1988 to the PEF laboratory at the FDA in the US. This transfer had four aims. The first was to link wild poliovirus whole genome sequences with case histories to improve our understanding of the epidemiology of the last AFP cases caused by wild poliovirus infections in Israel [3,5,17]. The second was to generate complete or near complete genome sequences of polioviruses isolated from a time period when few sequences were publicly available for any polioviruses isolated anywhere. The third was that it would be possible to destroy potentially infected stocks once sequenced while “preserving” them in silico for further study since viable polioviruses can be generated *de novo* [19]. The fourth was to provide proof of concept that, as we demonstrated for archived samples of another enterovirus, echovirus 11 [11], we could retrieve WGS data from archived stocks of poliovirus, without regard to viability and without the need to re-amplify them in cell culture.

The study, described here, describes the WGS of PV3 polioviruses isolated from stools of AFP cases and/or contacts. Seven distinct lineages of WPV3 were isolated from stools of AFP cases between 1973 and 1988 in Israel based on the phylogenetic analysis of sequences of their P1 regions (Figure 1) coupled with additional sequence information from their P2-P3 regions. Annual AFP rates ≥2 per 100,000 children <15 years old throughout this period (Table 1) indicate that, most likely, all AFP cases were investigated each year. The number and temporal pattern of cases with WPV3 isolates belonging to lineages B through G are consistent with sporadic cases, due to separate introductions and chains of transmission lasting a few years at most. In contrast, the 23_T2_._19_ AFP cases associated with isolates of lineage A that appeared for at least 6 years, starting in 1973, and clearly fit the pattern of an outbreak of prolonged duration. The duration of transmission may have been even longer if related cases occurred before 1973, the beginning of the period of observation. Moreover, members of all of the lineages may have been missed, since the only 43_T2_._13_ whole genomic sequences were recovered from 58_T2_._09_ cases with ≥1 archived WPV3 isolates (Table 2 and Table 4). 

Phylogenetic analysis of P1 sequences (Figure 2) suggested that three sub-lineages of Lineage A co-circulated. This was confirmed by SimPlot analysis

SimPlot analysis of isolates of the complete open reading frames (P1, P2, and P3 genomic regions) confirmed the three lineages and revealed that both the A2 and A3 sub-lineages could each be further subdivided into two sub-sub-lineages based on different the recombinations in non-structural genes in P2 and/or P3. Both of the P2-P3 region recombinant sequences of sub-lineage A3 were different from the P2-P3 regions of isolates in sub-lineages A1 and A2 and likely occurred after A3 diverged from A1 and A2. The years during which cases were associated with A3a (1976–1977) and of A3b (1976 to 1978) isolates imply that both circulated in Israel for some time. The three sub-lineages of lineage A may have diverged during endogenous circulation in Israel, although it is more likely that they represent multiple simultaneous importations from a common external reservoir, where they had diverged and recombined. If sequences from such an external reservoir had been available, we could have inferred multiple introductions, rather than divergence during local circulation, if the sequences of each of the two isolates showed higher similarity to the sequences in the reservoir than to each other (see Manor et. al. [20] for an example where such an analysis was successfully applied to two WPV1s isolated from sewage in Gaza, one in February and one in August in 2000).

Isolates from 11_T2_._14_ AFP cases were SL3s, rather than WPV3s, by sequence-based ITD. One_T2_._17_ of the SL3s was a minor component of a case where WPV3 was the major component. Such a large number of SL3s might be unexpected, since these were AFP cases. The association of the isolates with AFP may have been coincidental, given the short interval between onset of symptoms and a last dose of OPV in some of the cases (Table 6). However, it is possible that some of these SL3s contributed to or caused the AFP, since in 8_T2_._23_ of the isolates from the 11_T2_._14_ cases, SL3 had recombined with sequences from OPV1, OPV2 and/or a non-polio enterovirus (NPEV) (Figure 2), and in silico analysis indicated reversion to the neurovirulent genotype of some or all of the neurovirulence attenuation sites of OPV3. If they did contribute etiologically to the AFP, then in the absence of sequence confirmation, historical reports on the incidence of poliovirus-associated paralytic poliomyelitis (VAPP) may be an underestimation.

The clinical symptoms of cases with PV3-positive stools are listed in Table 7. Of potential interest is that cases associated with lineage A presented with paralysis and/or paresis, while the following symptoms were underrepresented, compared with the other AFP cases associated with PV3s: fever, rash, sore throat, and/or problem swallowing, diarrhea/nausea, respiratory/bulbar involvement, meningitis/encephalitis, unconsciousness, and general paresis. 

Seroconversion after a poliovirus infection is generally defined a change from a seronegative antibody titter of <1:8 before infection to a titter ≥8 after infection or a 4-fold rise in antibody titer for individuals who were seropositive before vaccination [21]. However, the Polio Laboratory Manual 4th Edition states that:

*“Testing for polio neutralizing antibodies is not recommended for routine use in the diagnosis of poliomyelitis. It has rarely been useful in clarifying questionable virus diagnoses and requires additional field and laboratory time. Interpreting serum antibody titres is difficult with widespread immunization, and the method does not differentiate between antibodies against wild and vaccine strains.”*

Many of the case reports included kinetic studies of neutralizing IgG antibody titers. 

These results, summarized in Table 8, confirm that seroconversion may be less useful for determining the etiology of a poliomyelitis case than serotyping the poliovirus isolated from the stools of the patient. However, vaccination with OPV prior to appearance of symptoms may confound the interpretation of results obtained by serotyping the poliovirus isolated from stools and confirmation by RT-PCR and WGS ITD. 

In conclusion, (1) we provide proof of concept that we can obtain WGS of archived neurovirulent polioviruses, independent of viability by sequencing RNA extracted directly from the archived material, e.g., without the need to grow high titter cultures of the viruses; (2) we provide sequence-based information on the epidemiology of circulation of wild polioviruses; and (3) we provide whole or near-whole genome sequence information about the wild polioviruses that circulated between 1973 and 1988 and related them to case reports of the AFP cases from which they were isolated.

## Figures and Tables

**Table 1:** Annual AFP incidence, AFP rates, and PV3-positive cases in Israel between 1973 and 1988.

Year	AFP Cases	Total Pop (×10^5^)	Pop ≤ 15 Yrs (×10^5^)	AFP Rate/(×10^5^) ≤15 Yrs	PV(+) AFP Cases	PV3(+) AFP Cases	PV(−) Cases PV3(+) Contacts	PV(+) PV3(−) Cases PV3(+) Contacts
1973	86	3338	935	9.2	26	13	2	3
1974	164	3422	958	17.1	69	8	0	0
1975	77	3493	978	7.9	38	11	1	2
1976	150	3575	1001	15	84	9	1	2
1977	76	3653	1023	7.4	25	5	0	0
1978	72	3738	1047	6.9	36	4	0	0
1979	77	3836	1074	7.2	39	4	0	0
1980	70	3922	1098	6.4	40	1	0	0
1981	42	3978	1114	3.8	10	2	0	0
1982	32	4064	1138	2.8	8	2	0	0
1983	35	4119	1153	3	10	4	0	0
1984	22	4200	1176	1.9	2	0	0	0
1985	23	4266	1194	1.9	6	1	0	0
1986	27	4331	1213	2.2	7	3	0	0
1987	12	4407	1234	1	4	1	0	0
1988	55	4477	1254	4.4	23	5	0	1
**Total**	**1020**				**427**	**73**	**4**	**8**

**Table 2:** Cases and isolates described in this manuscript.

ID	Number	Description
AFP Cases		
T2.01	1020	AFP cases investigated between 1973 and 1988
T2.02	427	AFP cases with PV(+) stools
T2.03	85	AFP cases with PV3(+) stools from the index case and/or contacts
T2.04	73	AFP cases with PV3(+) positive stools
T2.05	8	AFP PV(+)/PV3(−) cases with PV3(+) contacts
T2.06	4	AFP PV(−) cases with PV3(+) contacts
	Archived PV Isolates	
T2.07	257	Archived PV isolates (Types 1, 2, and 3)
T2.08	93	PV3(+) isolates reported for the index case and/or contacts (isolates from 93 individuals)
T2.09	71 of the 93	Archived PV3 isolates (isolates: 70 labeled PV3; 1 labeled PV1) from 58 cases with archived PV3(+) samples from the index case and/or contact
	Whole Genome Sequences (WGS)	
T2.10	55	PV3(+) isolates with WGS from 52 WPV3(+) cases and 1 WPV1(+) case
T2.11	53 of the 55	PV3(+) WGS from 51 WPV3(+) cases and 1 WPV1(+) case
T2.12	2 of the 55	WGS available from PV3(-), PV(+) cases
T2.13	43 of 55	WPV3(+) WGS from 42 archived isolates from 34 cases
T2.14	12 of 55	SL3(+) WGS from 11 archived isolates from 11 cases—one sample contained two isolates
T2.15	9 of 12	Isolates from VP3(+) cases (including 2 isolates from one case)
T2.16	1 of 12	Isolate from a case with a mixture of PVs
T2.17	1 of 12	Isolate from a case with a major incomplete WPV3 WGS component
	Genetic lineages	
T2.18	7	Lineages (arbitrarily labeled A to G)
T2.19	23	Isolates with WGS from Lineage A
T2.20	15	Isolates in sub lineage A1
T2.21	2	Isolates in sub lineage A2
T2.22	6	Isolates in sub lineage A3
T2.23	8	SL3 ^a^ with recombinant genomes

^a^ SL3 = Sabin-like 3—a type 3 polio isolate with a viral capsid protein gene sequence that has diverged from the sequence of the Sabin 3 vaccine seed strain sequence by <1%.

**Table 3:** Demographic data for cases and/or contacts with PV3-positive stools.

	PV3(+) Cases and Contacts ^a^ (*n* = 93_T2_._08_)	PV3(+) Case ^b^ (*n* = 73_T2_._04_)
Ethnicity		
Jewish	23	16
Non-Jewish (Arab)	69	56
Non-Jewish (Druse)	1	1
Not listed	0	0
Geographical location		
Israel (all health districts)	39	26
West Bank/E. Jerusalem	5	4
Gaza	45	41
Not listed	3	2
Other (Jordan)	1	0
Gender		
Male	56 (60%)	47 (64%)
Female	33 (36%)	23 (32%)
Not recorded/not clear	4 (4%)	3 (4%)

^a^ cases and/or contacts with PV3(+) stools, plus PV3(−) cases with ≥1 PV3(+) contact with PV3(+) stools. ^b^ cases with PV3(+) stools.

**Table 4:** Isolates from cases and/or contacts, from which complete P1 PV3-positive sequences were obtained by WGS.

Description of Archived Sample	Number Archived	Number Sequenced		
		Total	WPV3	SL3
Isolates from Cases				
PV3-positive	53_T2_._11_	43_T2_._13_	33	10 ^a^
PV3-positive and PV1-positive	2	1	0	1
PV3-positive and PV2-positive	1	1	1	0
PV3-positive and PV1- and PV2-positive	2	0	0	0
Isolates from PV3-positive Contacts				
PV-negative cases	4	1	1	0
PV1-positive cases	3	0	0	0
PV2-positive cases	2	0	0	0
PV3-positive cases	7	6	6	0
Anomalous result				
PV3 sequence from a PV1-positive case	1	1	1	0

^a^ Ten cases with 11 isolates—two closely related SL3 sequences were obtained from one archived isolate.

**Table 5:** In silico analysis of reversion to neurovirulence of SL3 isolates.

	5′UTR	VP3	VP1	OPV Doses	Interval between Last OPV and Symptoms	Recombinant
Nucleotide Substitution	U_472_C	U_2034_C	U_2493_C			
Amino Acid Substitution	*n*/a	phe_91_ser	thr_6_iso			
SL3_ISR-1978-04	C	phe	iso	1	<30 days	No
SL3_ISR-1978-08	U_472_C	ser	iso	1	18 days	Yes
SL3_ISR-1978-33	U_472_C	phe	iso	1	1 day	Yes
SL3_ISR-1980-03a	U_472_C	phe	iso	0	unvaccinated	Yes
SL3_ISR-1982-02	U_472_C	ser	iso	3	176 days	Yes
SL3_ISR-1983-05	U_472_C	ser	iso	1	44 days	Yes
SL3_ISR-1983-06	U_472_C	phe	iso	2	8 days	Yes
SL3_ISR-1987-00	U_472_C	phe	iso	1	<30 days	No
SL3_ISR-1988-13	U_472_C	phe	iso	Unk	Years	Yes
SL3_ISR-1988-15	U_472_C	phe	iso	2	7 days	Yes
SL3_ISR-1988-25	U_472_C	ser	iso	1	14 days	No

Grey highlight indicates reversion site unchanged. <30 days = exact dates not recorded. Unk = 32 years old, vaccinated but not recorded.

**Table 6:** The OPV vaccination histories of cases with WPV3-positive and SL3-positive stool isolates.

OPV Vaccination History						
	PV3(+) (*n* = 85_T2_._03_)	PV3(+) Case ^a^ (*n* = 73_T2_._04_)	PV3(+) Cases with Sequenced Isolates			
			PV3(+) (*n* = 43_T2_._13_)	WPV3(+) A1 (*n* = 15_T2_._20_)	WPV3(+) A3 (*n* = 6_T2_._22_)	SL3(+) (*n* = 11_T2_._14_)
Unvaccinated ^b^	18 ^b^	17 ^b^	9	3	1	1
1 dose OPV	18	13	8	3	0	6 ^c^
2 doses OPV ^d^	8	7	3	0	0	2
3 doses OPV ^e^	23	21	12	9	1	1
4 doses of OPV	12	9	7	0	4	0
Not recorded or incomplete ^f^	6	6	4	0	0	1
**Days between last OPV dose and appearance of symptoms**						
	**PV3(+) (*n* = 55_T2_._10_/85_T2_._03_)**	**PV3(+) case ^b^** **(*n* = 43_T2_._13_/73_T2_._04_)**	**PV3(+) Cases with Sequenced** **Isolates ^c^**			
			**PV3(+)** **(*n* = 30/43_T2_._13_)**	**WPV3(+)** **A1 (*n* = 12/15_T2_._20_)**	**WPV3(+)** **A3 (*n* = 5/6_T2_._22_)**	**SL3(+)** **(*n* = 6/11_T2_._14_)**
Average	219	244	175	164	243	45
Median	113	130	131	155	331	16
Minimum	3	7	7	24	24	7
Maximum	2006	2006	968	355	421	176
Number < 30 days	19	13	8	2	1	5

^a^ AFP cases with PV3(+) stools. Does not include PV3(−) patients with PV3(+) contacts and PV(+)/PV3(−) patients with PV3(+) contacts. ^b^ One child received 1 dose of IPV only. ^c^ Includes one case with WPV3 partial sequence. ^d^ One child received two doses of IPV plus two doses of OPV. ^e^ One child received 3 doses of OPV plus one dose of monovalent type 1OPV. ^f^ Children with no vaccination history recorded or who were vaccinated, but details of vaccine type and number of doses were not recorded.

**Table 7:** Summary of clinical symptom associated with PV3-positive cases.

Symptom		PV3(+) (*n* = 85_T2_._03_)	PV3(+) Cases ^a^ (*n* = 73_T2_._04_)	Sequence Confirmed Cases			
				PV3(+) (*n* = 43_T2_._13_)	WPV3(+) A1 (*n* = 15_T2_._20_)	WPV3(+) A3 (*n* = 6_T2_._22_)	SL3 (*n* = 10_T2_._14_) ^b^
Fever		14	12	0	0	0	0
Rash		1	1	0	0	0	0
Sore throat or problem swallowing		2	2	0	0	0	0
Diarrhea/nausea		2	2	0	0	0	1
Respiratory/bulbar		4	4	3	1	0	1
Meningitis/encephalitis		3	2	0	0	0	1
Unconscious/coma		2	2	1	1	0	0
Death		3	3	0	0	0	1
General paresis		22	21	9	2	1	3
	Left leg only	6	6	5	1	1	0
	Right leg only	2	2	0	0	0	0
	Left and right legs only	3	3	3	1	0	1
	Left arm only	0	0	0	0	0	0
	Right arm only	2	2	0	0	0	0
	Left and right arm only	0	0	0	0	0	0
	Arms and legs (any combination)	7	6	1	0	0	1
	One leg (not designated)	2	2	0	0	0	1
General paralysis		56	44	25	12	4	5
	Left leg only	23	21	13	6	4	1
	Right leg only	6	4	2	1	0	1
	Left and right legs only	17	15	10	5	0	2
	Left arm only	0	0	0	0	0	0
	Right arm only	0	0	0	0	0	0
	Left and right arm only	0	0	0	0	0	0
	Arms and legs (any combination)	10	4	0	0	0	0
Misc (palsy, arthritis, GB, otitis, facialis)		4	4	2	0	0	2

^a^ AFP cases with PV3(+) stools. Does not include PV3(−) patients with PV3(+) contacts and PV(+)/PV3(−) patients with PV3(+) contacts. ^b^ Data available for 10 of the 11 cases.

**Table 8:** The number of sequence-confirmed, PV3-positive cases with >1:8 anti-PV3 IgG titers, <1:8 titers of anti-PV2 IgG and <1:8 titers of anti-PV3 IgG, and cases that seroconverted or had a ≥4-fold increase in anti-PV3 IgG titters.

Lineage		PV3 Total (*n* = 43_T2_._13_)	Positive ^a^ or ≥4-Fold Increase in Anti-PV3 IgG			>Anti-PV3 IgG or ≥4-Fold Increase in IgG		Not Done or Not Recorded
			Positive But No Increase ^a^	Sero-Conversion or ≥4-Fold Increase	Total	Anti-PV1 IgG	Anti-PV2 IgG	
A		23	5	9 ^b^	11 ^b^	2	1	6
	A1	15	5	6 ^b^	11 ^b^	2	0	1
	A2	2	0	0	0	0	0	2
	A3	6	0	3	3	0	1	3
B		3	1	2	3	1 ^c^	0	0
C		3	0	3	3	0	0	0
D		1	0	0	0	0	0	1
E		1	0	1	1	0	0	0
F		2	1	1	2	0	0	0
G		1	1	0	1	1 ^d^	0	0
SL3		11 ^e^	0	3	3	0	3	2
	SL3	3	0	1	1	0	1	1
	SL3_rec_	8 ^e^	0	2	2	0	3	1
PV3-positive Not sequenced		30	2	11	13	6 ^f^	9 ^g^	7

^a^ Anti-PV3 >1:8; anti-PV1; and anti-PV2 both <1:8. ^b^ In one case, the endpoint titers for anti-PV3 were ≥1:16,384, and the patients had 1:1024 titers against the other two serotypes. ^c^ One isolate PV3-positive by NGS was recorded as PV1- and PV3-positive and had >4-fold increases in anti-PV1 (1:256–>1:16,384) and anti-PV3 antibodies (<1:4–>1:4096). ^d^ O < PV1 (1:4096); anti-PV2 (>1:16,384); anti-PV3(1:16,384). ^e^ one SL3 was the minor component with a WPV3 as the major component. ^f^ One isolate had a 1:16,384 titter against PV1, seroconverted to 1:16,384 for anti-PV2, and was only 1:256 for anti-PV3, another seroconverted (1:4096–>1:10,384) for anti-PV1 and was less than 1:8 for anti-PV3. ^g^ 3 cases had ≥1:16,384 anti-PV2 titers and lower anti-PV3 titers and in a 4th case both anti-PV2 and PV3 were ≥1:16,384.

## Data Availability

Sequences were deposited in GenBank/EMBL/DDBJ (Access numbers OP137272 to OP137324.

## References

[1] Plotkin S.A., Vidor E., Plotkin S.A., Orenstein W.A., Offit P.A. (2008). Poliovirus vaccine-inactivated. Vaccines.

[2] Sutter R.W., Kew O.M., Cochi S.L., Plotkin S.A., Orenstein W.A., Offit P.A. (2008). Poliovirus vaccine-live. Vaccines.

[3] Swartz T.A. (2008). The Epidemiology of Polio in Israel an Historical Perspective.

[4] Shulman L.M., Meyers R.A., SpringerLink (Online service) (2020). Polio and Its Epidemiology. Encyclopedia of Sustainability Science and Technology Vol. Infectious Diseases.

[5] Slater P.E., Orenstein W.A., Morag A., Avni A., Handsher R., Green M.S., Costin C., Yarrow A., Rishpon S., Havkin O. (1990). Poliomyelitis outbreak in Israel in 1988: A report with two commentaries. Lancet.

[6] GPEI (2015). Global Eradication of Wild Poliovirus Type 2 Declared. http://www.polioeradication.org/mediaroom/newsstories/Global-eradication-of-wild-poliovirus-type-2-declared/tabid/526/news/1289/Default.aspx.

[7] WHO Two out of Three Wild Poliovirus Strains Eradicated. Global Eradication of Wild Poliovirus Type 3 Declared on World Polio Day. 24 October 2019. https://www.who.int/news-room/feature-stories/detail/two-out-of-three-wild-poliovirus-strains-eradicated.

[8] Previsani N., Tangermann R.H., Tallis G., Jafari H.S. (2015). World Health Organization Guidelines for Containment of Poliovirus Following Type-Specific Polio Eradication-Worldwide. MMWR Morb. Mortal. Wkly. Rep..

[9] WHO (2015). WHO Global Action Plan to Minimize Poliovirus Facility-Associated Risk after Type-Specific Eradication of Wild Polioviruses and Sequential Cessation of Oral Polio Vaccine Use (GAPIII). http://www.polioeradication.org/Posteradication/Certification.aspx.

[10] Metzker M.L. (2010). Sequencing technologies-the next generation. Nat. Rev. Genet..

[11] Laassri M., Zagorodnyaya T., Hassin-Baer S., Handsher R., Sofer D., Weil M., Karagiannis K., Simonyan V., Chumakov K., Shulman L. (2018). Evolution of echovirus 11 in a chronically infected immunodeficient patient. PLoS Pathog..

[12] Simonyan V., Mazumder R. (2014). High-Performance Integrated Virtual Environment (HIVE) Tools and Applications for Big Data Analysis. Genes.

[13] Karagiannis K., Simonyan V., Chumakov K., Mazumder R. (2017). Separation and assembly of deep sequencing data into discrete sub-population genomes. Nucleic Acids Res..

[14] Larkin M.A., Blackshields G., Brown N.P., Chenna R., McGettigan P.A., McWilliam H., Valentin F., Wallace I.M., Wilm A., Lopez R. (2007). Clustal W and Clustal X version 2.0. Bioinformatics.

[15] Sayers E.W., Beck J., Bolton E.E., Bourexis D., Brister J.R., Canese K., Comeau D.C., Funk K., Kim S., Klimke W. (2021). Database resources of the National Center for Biotechnology Information. Nucleic Acids Res..

[16] Kew O.M., Mulders M.N., Lipskaya G.Y., de Silva E., Pallansch M.A. (1995). Molecular Epidemiology of Polioviruses. Semin. Virol..

[17] Shulman L.M., Handsher R., Yang C.F., Yang S.J., Manor J., Vonsover A., Grossman Z., Pallansch M., Mendelson E., Kew O.M. (2000). Resolution of the pathways of poliovirus type 1 transmission during an outbreak. J. Clin. Microbiol..

[18] GPEI (2016). Classification and Reporting of Vaccine-Derived Polioviruses (VDPV) GPEI Guidelines. http://polioeradication.org/wp-content/uploads/2016/09/Reporting-and-Classification-of-VDPVs_Aug2016_EN.pdf.

[19] Cello J., Paul A.V., Wimmer E. (2002). Chemical synthesis of poliovirus cDNA: Generation of infectious virus in the absence of natural template. Science.

[20] Manor Y., Blomqvist S., Sofer D., Alfandari J., Halmut T., Abramovitz B., Mendelson E., Shulman L.M. (2007). Advanced environmental surveillance and molecular analyses indicate separate importations rather than endemic circulation of wild type 1 poliovirus in Gaza district in 2002. Appl. Environ. Microbiol..

[21] Giri S., Kumar N., Dhanapal P., Venkatesan J., Kasirajan A., Iturriza-Gomara M., John J., Abraham A.M., Grassly N.C., Kang G. (2018). Quantity of Vaccine Poliovirus Shed Determines the Titer of the Serum Neutralizing Antibody Response in Indian Children Who Received Oral Vaccine. J. Infect. Dis..

